# Diastolic Heart Failure Mechanisms and Assessment Revisited

**DOI:** 10.3390/jcm13113043

**Published:** 2024-05-22

**Authors:** Ramdas G. Pai, Padmini Varadarajan

**Affiliations:** 1 Department of Medicine, University of California Riverside School of Medicine, Riverside, CA 92521, USA; padrav2001@yahoo.com; 2 Department of Medicine, California University of Science and Medicine, Colton, CA 92324, USA

**Keywords:** diastolic heart failure, HFpEF, relaxation, stiffness, compliance, echocardiography, Doppler

## Abstract

The syndrome of heart failure (HF) with preserved ejection fraction (HFpEF) makes up about half of the HF population. The HF mechanisms in these patients are varied and not fully understood. In addition, the term “diastolic HF” was switched to HFpEF because of difficulties in measuring the left ventricular (LV) diastolic performance. In the late stages, HFpEF carries a prognosis that is as bad as or worse than that of HFrEF. Hence, it is important to recognize LV diastolic impairment at an earlier stage so that the causal mechanisms, if any, can be treated to retard its progression. Despite the availability of numerous disease-modifying agents for HFrEF, there are hardly any available treatments for HFpEF. With our aging population, there will be an epidemic of HFpEF and hence this entity needs attention and respect. In this paper, we review the fundamental mechanisms of HFpEF, the physiology of LV filling and how LV diastolic function can be comprehensively measured. We also speculate how this may help with the early recognition of diastolic HF and its treatment.

## 1. Introduction

**Figure 1:**
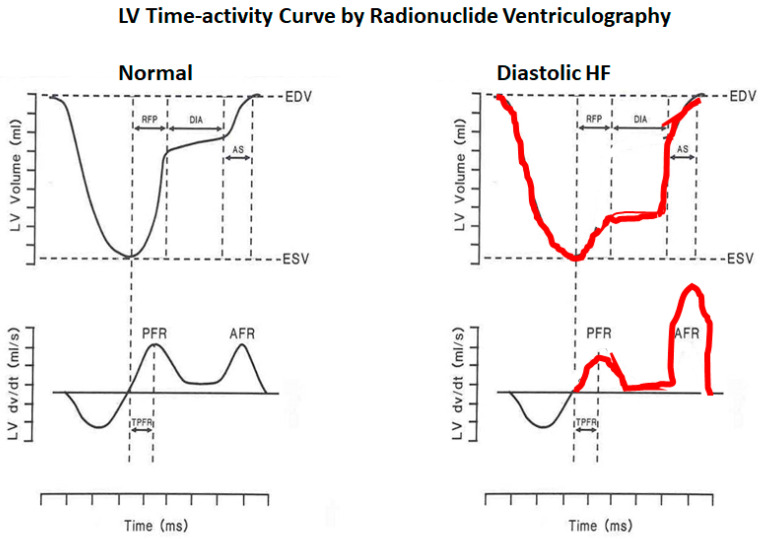
LV time–activity curve by the radionuclide technique in a typical patient with diastolic heart failure and a normal control: note the reduced early filling and filling rate and the reliance on atrial systole in the patient with diastolic heart failure. AFR = Atrial filling rate; AS = Atrial systole; DIA = Diastasis; EDV = End-diastolic volume; ESV = End-systolic volume; PFR = Peak filling rate; RFP = Rapid filling period; TPFR = Time to peak filling rate.

**Figure 2:**
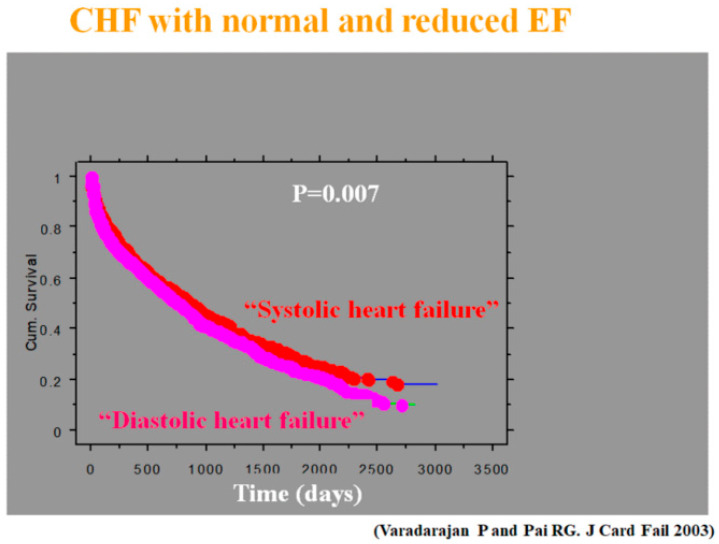
Prognosis of heart failure (HF) patients admitted to the hospital for decompensation [5]: though both groups fared poorly, the survival for diastolic heart failure was worse. CHF = Congestive HF; EF = Ejection fraction.

The fact that heart failure (HF) can occur despite a normal left ventricular (LV) ejection fraction (EF) was first described in the early 1980s [1,2]. It was observed in these patients, based on radionuclide ventriculography showing LV time–volume curves, that early LV filling was impaired, with a greater contribution from atrial contraction, and the term “diastolic heart failure” was coined (Figure 1). Subsequently, this was changed to HF with preserved EF (HFpEF) because of the complexities of the measurement of LV diastolic function [3]. Diastolic HF was initially thought to be associated with a better prognosis compared to systolic HF based on Framingham data [4], but it was subsequently shown to be as sinister as or worse than systolic HF, especially in hospitalized class IV patients with HF (Figure 2) [5]. We would like to summarize the growth in the understanding of LV diastolic function (Table 1).

However, it has to be recognized that clinical HF is a late stage of the disease process. It is important to recognize early abnormalities in LV diastolic function. Diastole is more vulnerable than systole to a variety of disease processes and insults. Hence, early recognition of LV diastolic dysfunction may provide earlier insights into myocardial processes that may eventually produce HF. This opens up opportunities to treat the disease in the early stages and potentially prevent progression to clinical HF and reduce the HF burden in the community. Some of the examples of this include hypertensive heart disease and infiltrative disorders such as cardiac sarcoidosis and amyloidosis. Many other pathophysiological bases of diastolic HF are poorly understood.

**Figure 3:**
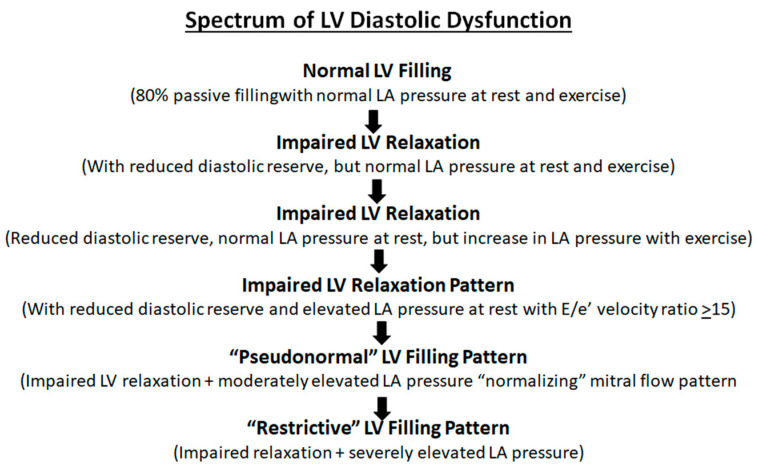
Spectrum of left ventricular (LV) diastolic dysfunction: proposed progression from normal diastolic function to progressive impairment of LV relaxation and compliance with clinical worsening at each stage. LA = Left atrium.

In this review, we will cover the spectrum of LV diastolic dysfunction (Figure 3), physiology of diastole, factors affecting LV diastolic function and measurement of LV diastolic function.

## 2. Physiology of Diastole

**Figure 4:**
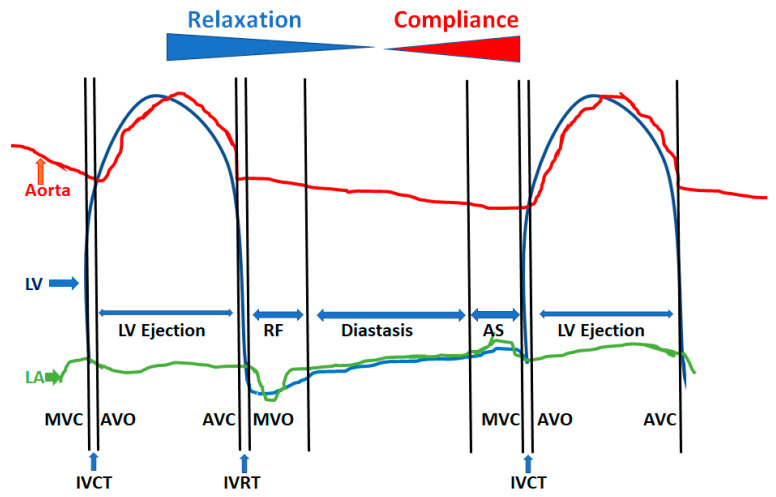
Phases of diastole and their determinants. The 4 phases of diastole are shown: left ventricular (LV) isovolumic relaxation phase and time (IVRT), which starts with aortic valve closure (AVC) or A2 and extends to mitral valve opening (MVO); phase of rapid filling (RF), which is the early passive filling of the LV marked by the mitral E wave and is heavily dependent upon LV relaxation and recoil; diastasis, where less than 5% of LV filling normally occurs and is the phase of diastolic reserve that is used up at faster heart rates; and atrial systole (AS), which is the booster pump to achieve maximal LV filling in the end-diastole volume without having a need to increase the left atrial (LA) pressure throughout diastole. The latter part of diastole is dependent on the LV compliance and atrial systolic function. AVO = Aortic valve opening; IVCT = LV isovolumic contraction time; MVC = Mitral valve closure.

Diastole starts with aortic valve closure (A2 component of S2) and extends to the LV isovolumic contraction phase. It is made of four distinct phases (Figure 4).

1. LV isovolumic relaxation from aortic valve closure to mitral valve opening
2. Early phase of rapid passive filling
3. Diastasis
4. Atrial systole

There are multiple processes that control or affect different phases of diastole, and they are described below (2).

### 2.1. LV Relaxation Process

The early part of diastole is determined by the process of active LV relaxation, which is an energy-dependent process and is the reversal of the contractile process. It starts in late systole and extends into the early LV filling phase. In patients with hypertrophic cardiomyopathy, where relaxation is very abnormal, it can extend further—occasionally resulting in a fall in the LV diastolic pressure into mid-diastole despite LV filling [6]. It depends not only on the active relaxation of individual muscle fibers but also on the spatial and temporal synchrony of relaxation [7,8]. Asynchrony would impair the rate of LV pressure decay. Factors affecting the LV relaxation process are listed in Table 2.

### 2.2. Early Diastolic Suction Due to LV Recoil

This sort of suction force is especially prominent in mitral stenosis, severe volume loss and hypercontractile ventricles such as hypertrophic cardiomyopathy. The suction force would facilitate early diastolic filling. This is one of the reasons why, in hyperdynamic LVs, even with LV hypertrophy, the early passive filling could be normal despite markedly impaired LV relaxation—a phenomenon frequently seen in hypertrophic cardiomyopathy. When the LV volume is very small because of excessive shortening of the muscle fibers, their springing back into the resting state generates recoil. The recoil of the LV twist may contribute to this phenomenon as well.

### 2.3. Left Ventricular Compliance

Compliance is the ratio of change in volume over pressure or the amount of increase in volume per unit increase in distending pressure in the passive stage. The reciprocal of this or the amount of increase in pressure per unit change in volume is stiffness. Compliance comes into play in the latter half of LV diastole [9,10]. Its measurement will be described later. Compliance can decrease with age, LV hypertrophy, fibrosis, infiltrative disorders, etc. (Table 3) [11].

### 2.4. Myocardial Stiffness

This is measured as the modulus of myocardial stiffness through the stress–strain relationship, as described later, and measures the passive elastic properties of muscle fibers corrected for geometry. This is a direct measure of the passive properties and is increased in muscle fiber hypertrophy, fibrosis and infiltration.

### 2.5. Erectile Effects of Coronary Filling

Because of the squeezing of intramyocardial coronaries during systole, epicardial coronaries store blood and energy during this phase, which is dissipated in diastole. This is thought to aid in early diastolic LV relaxation and filling.

### 2.6. Factors Affecting Pericardium or Intrapericardial Pressure

The normal intrapericardial pressure is the same as the atmospheric pressure. Hence, the transmural LV diastolic pressure, which is the true LV-filling pressure, is the same as the cavitary LV diastolic pressure. For example, if the LV end-diastolic pressure (LVEDP) is 15 mmHg, the transmural LV diastolic pressure is 15 mmHg. It is not so if the intrapericardial pressure is higher. For example, if the intrapericardial pressure is 15 mmHg, as may happen in cardiac tamponade, to produce the same transmural LV-filling pressure, the LVEDP has to be 30 mmHg to achieve equivalent LV filling. The intrapericardial pressure is elevated not only in cardiac tamponade but also in pericardial constriction, situations in which pericardial constraint is invoked and situations where the mediastinal pressure is elevated. The pericardial constraint is invoked when there is an acute increase in the volume of any of the cardiac chambers such that the unyielding pericardium would cause an increase in the intrapericardial pressure. Examples of this include acute valve leaks because of infection, trauma or infarction causing acute regurgitation of mitral/aortic/tricuspid valves, right ventricular infarction or acute massive pulmonary embolism where the right ventricle dilates because of dysfunction or acute afterload mismatch. This may also occur in acute pericarditis because of edema and cellular infiltrates in the pericardium making it stiffer. The conditions that increase the mediastinal pressure, which is transmitted to the pericardium, include large pleural effusions, massive tumors, tension pneumothorax and use of positive end-expiratory pressure (PEEP) ventilation. When PEEP is used, or if auto PEEP occurs because of expiratory obstruction, about 50% of the raised intrapleural pressure is transmitted to the pericardial space. Hence, it is important to remember to factor PEEP into the interpretation of hemodynamics obtained invasively in the intensive care unit. These conditions may mimic LV diastolic failure and must be kept in mind as differential diagnoses as its mimickers (Table 4).

### 2.7. Atrial Systolic Function

Late diastolic LV filling is caused by an atrial booster pump. It accomplishes what early diastole may not do in terms of the optimal ventricular filling and preload. If early diastolic filling is impaired because of impaired LV relaxation, atrial systole compensates for that with enhanced booster function (e.g., older individuals) and vice versa. If early filling is efficient because of supernormal LV relaxation, resulting in exaggerated early LV filling (resulting in physiological S3) as occurs in young children, the atrial contribution will be minimal and such patients can tolerate very high heart rates without hemodynamic consequences as LV filling occurs rapidly and adequately in the first 100 ms of diastole. The relationship between LV early filling (mitral E wave) and the atrial contribution can be compared to that between a medical intern and a resident! If the intern is efficient, the resident has to do very little. If the intern is bad, the resident (atrial systole) has to work hard to compensate. If both are bad, there is trouble and the attending (elevated left atrial pressure) has to come into play!!! The atrial transport function and volume depend upon three things according to the Sarnoff model: atrial preload or state of the left atrial filling, atrial afterload or the pre-A left ventricular diastolic pressure and atrial mechanical function [12]. For example, if the LV diastolic pressure is high, the left atrial afterload increases, resulting in reduced left atrial ejection. Examples of reduced left atrial contractility include atrial myopathy and immediate post-cardioversion state.

### 2.8. Heart Rate, Diastolic Filling Rate and Diastolic Stress

**Figure 5:**
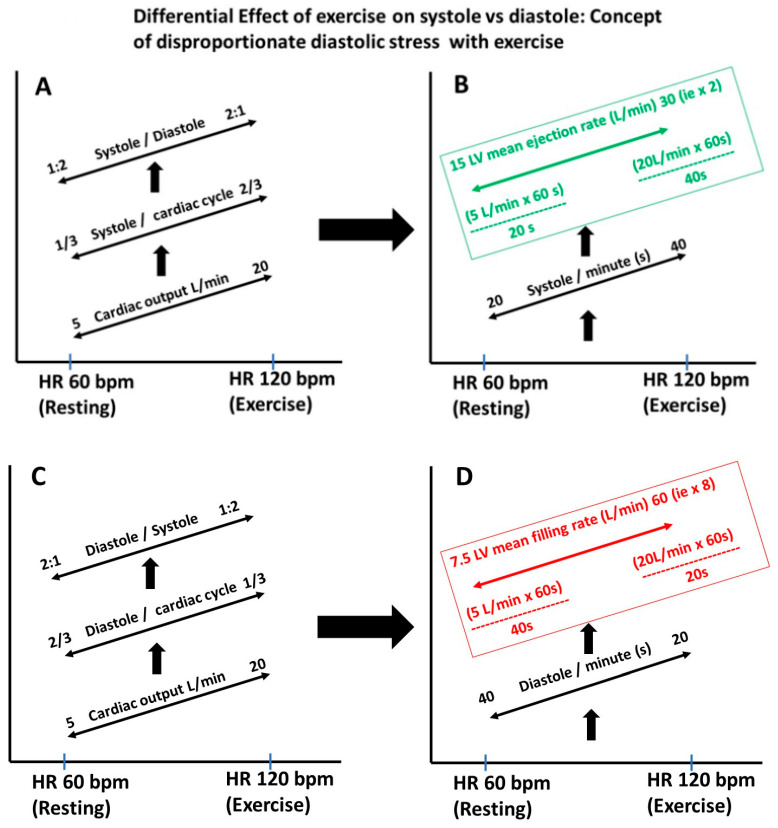
(**A**–**D**) Differential stress on diastole vs. systole when the heart rate is increased. During exercise, the cardiac output increases as a result of the increase in the heart rate (HR) and stroke volume. In this example, the resting HR of 60 bpm and cardiac output of 5 L/min increased to 120 bpm and 20 L/min, respectively. At rest, diastole occupies about 2/3 of the cardiac cycle, and with exercise, only 1/3 of the cardiac cycle, placing disproportionate stress on LV filling and requiring a higher filling rate than the LV ejection rate. In this example, the ejection rate just had to double, but the mean filling rate had to increase by 8 folds, stressing the need for diastolic efficiency and why even early LV disease will manifest because of LV dysfunction.

An increase in the heart rate stresses systole and diastole differentially, placing an excessive burden on diastole, as shown in Figure 5. This is because of the disproportionate shortening of diastole compared to systole, as explained in the figure legend. This is one of the reasons why diastole is more sensitive than systole to a variety of myocardial insults and why diastolic dysfunction is an early manifestation of disease. This underscores the importance not only of recognizing HFpEF but also of being able to pick up on the early signs of deranged LV diastolic mechanisms. Diastolic stress may be able to uncover covert LV diastolic dysfunction.

### 2.9. Functional and Structural Basis of Diastolic Impairment

As we alluded to before, it is also important to identify the underlying mechanisms of LV diastolic dysfunction as it is a syndrome consisting of clinical HF with preserved EF. Many of the underlying mechanisms may be reversible. Examples include treating blood pressure to reduce the LV afterload to promote relaxation and reduce myocardial energy expenditure, relieving obstruction in hypertrophic obstructive cardiomyopathy for similar reasons, treatment of ischemia, addressing LV dyssynchrony if possible, looking for myocardial infiltrative disorders, especially cardiac amyloid, with Tc-PYP scans, cardiac MRI and serological testing (as amyloid is treatable), looking for cardiac sarcoid when there are other features, etc.

## 3. Assessment of Diastole

### 3.1. Invasive Measure

There are three fundamental invasive measures of LV diastolic function. These are (1) the measures of LV early diastolic relaxation measured as Tau, (2) the measure of LV late diastolic stiffness measured as the modulus of chamber stiffness (k), and (3) the modulus of myocardial stiffness. The first two affect LV cavitary behaviors and the last one is the passive property of the muscle fiber derived from the LV stress–strain relationship.

#### 3.1.1. Measurement of LV Relaxation

**Figure 6:**
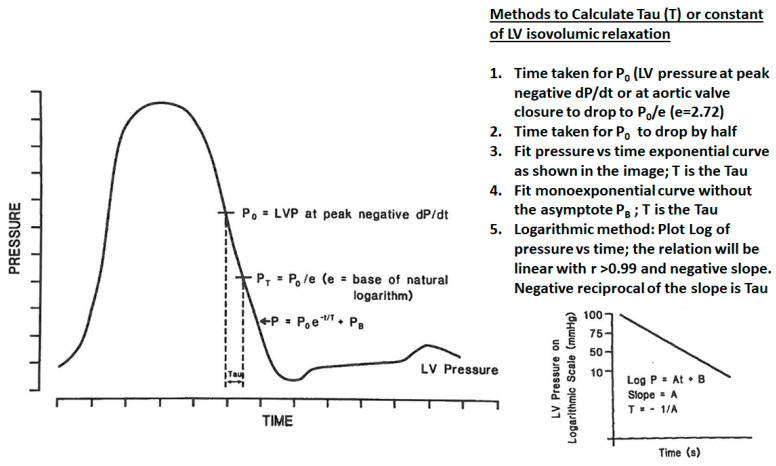
Measurement of Tau: Tau is a mathematical description of the rate of LV pressure decay after aortic valve closure or peak negative dP/dt. Various methods of computing it are listed in the figure, but the monoexponential method is the standard one. It has to be kept in mind that the pressure tracings should be of a high quality, with a high-frequency response system, and this is typically obtained by micromanometer tipped catheters, which have a frequency response of 200 Hz.

This is fundamentally described by Tau, which mathematically describes the rate of LV pressure decay after aortic valve closure or following peak negative dP/dt. There are multiple ways researchers have fitted equations to this pressure–time curve, but the fit has to be perfect, with a correlation coefficient of >0.99. Figure 6 summarizes some of these methods. The peak negative dP/dt is another measure that is used as a measure of LV relaxation, but it is a one-point measure rather than covering the entire duration of pressure decay and is less desirable. Typically, the normal peak negative dP/dt is in the range of −1500 mmHg/s and Tau, depending on the method used, is 30–60 ms. The longer the Tau, the worse the LV early diastolic relaxation.

#### 3.1.2. Measurement of Passive Elastic Properties of LV

**Figure 7:**
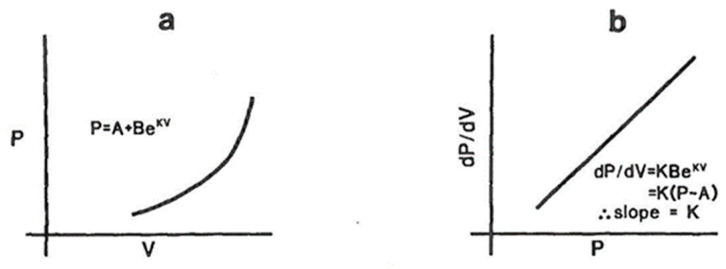
Measurement of LV compliance. (**a**) As the LV fills passively in the latter stage of diastole, the rate of the pressure rise depends on the degree of filling; hence, instantaneous compliance is volume-dependent. The pressure (P)–volume (V) relationship is exponential, as shown in (**a**). A and B are constants and K is referred to as the modulus of LV chamber stiffness. K is a number that is volume-independent and fundamentally describes the viscoelastic properties of the LV. To obtain this, you need a simultaneous recording of the LV diastolic pressures with a micromanometer tipped catheter (not fluid-filled, which has a frequency response <10 Hz) and LV volumes by contrast angiography at 60 fps or obtained by conductance catheter coupled with the micromanometer pressure tip. (**b**) As the instantaneous rate of the pressure rise (dP/dV) depends on the instantaneous volume and/or pressure, mathematics from (**a**) shows that the relationship between diastolic dP/dV and P is linear and the slope of this linear equation is the modulus of chamber stiffness, K.

As the LV fills passively, the pressure rises in a curvilinear fashion, with the rate of the pressure rise being higher at a higher level of filling. This behavior is best described by fitting an exponential equation, as shown on Figure 7. At higher rates of filling, the LV viscous properties may come into play as well—i.e., the pressure rise may be higher at the instant of rapid filling because of the slower relaxation of the muscle fibers, which is attributable to the viscous properties of the LV. This may come into play during atrial systole or whenever the LV fills very rapidly. In other words, the dynamic compliance may be lower than the static compliance.

#### 3.1.3. Measurement of Myocardial Stiffness

The modulus of myocardial stiffness is obtained by using the stress–strain relationship. Stress is the deforming force per unit area and can be computed given the LV diastolic pressures, wall thickness, length and using geometric assumptions about LV cavity. Strain is the deformation produced by stress and is a passive property. With the advent of speckle-tracking echocardiography, it has become easier to compute both the regional and global LV strain, both in the long axis and in other directions. Because of the limited application and the complexity of its derivation, we will not discuss this any further.

Table 5 lists some commonly used terminology in this area with the meaning to help readers understand the literature better.

### 3.2. Echo-Doppler Techniques

In practice, echocardiography is the cornerstone of assessing LV diastolic function and filling pressures, and to some extent, gaining insights into the myocardial structure [13,14,15]. The tools available include the transmitral flow, pulmonary vein flow, Doppler tissue velocities of the mitral annulus, mitral E and A wave propagation inside the LV, velocity profiles or mitral and aortic regurgitation signals, left atrial size and dynamics, ultrasound elastography and myocardial backscatter analysis, etc.

#### 3.2.1. Transmitral Flow

**Figure 8:**
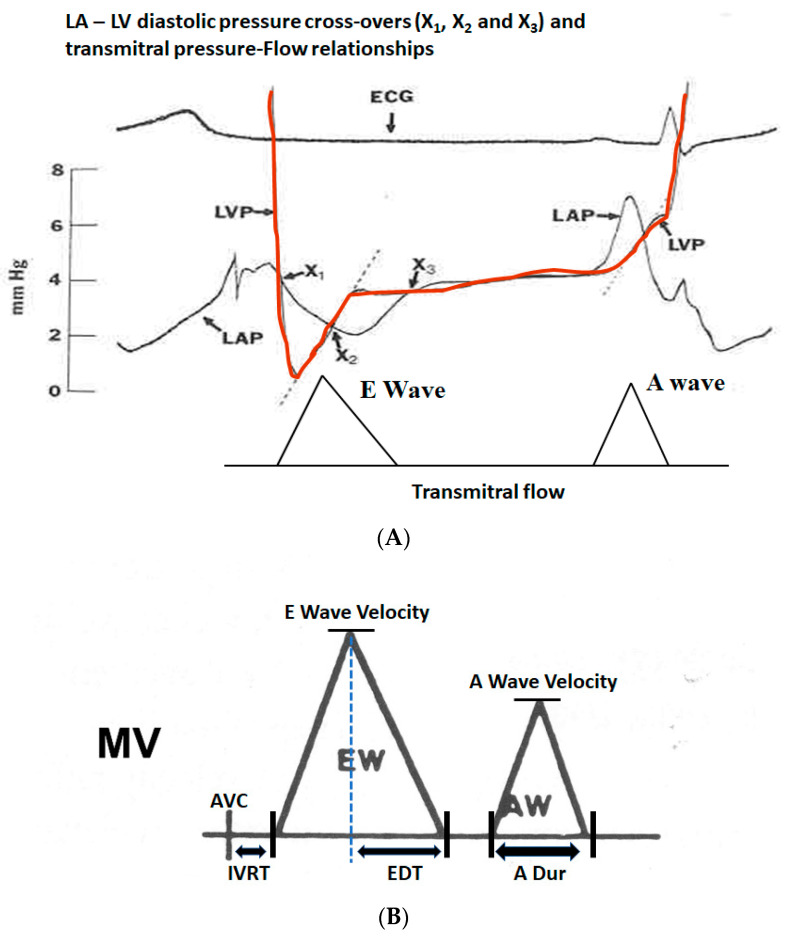
(**A**) Transmitral pressure–flow (P–F) relationships are poorly understood and the simplified Bernoulli equation does not apply in this situation as the pressure gradient is mainly used to accelerate the flow and the negative pressure gradient to decelerate the flow. When the LV pressure (red) drops below the LA pressure (black) at pressure cross-over X1, the transmitral flow starts and the gradient basically accelerates the transmitral flow. As the LV fills and the pressure in the LV equals that in the LA, X2 occurs when the pressure gradient is zero, but the E wave velocity is at the peak despite a zero pressure gradient. Between X2 and X3, the LV pressure is higher than that of LA and this negative gradient retards the mitral flow, resulting in its deceleration. It can be inferred from this P–F relationship that if the LV relaxation is slower, both the positive and negative gradients would be lower and the pressure cross-overs would be delayed, resulting in the slower acceleration of E wave, lower E wave amplitude and slower E wave deceleration, resulting in longer mitral E wave acceleration and deceleration times as well. On the other hand, higher LA pressure or very efficient LV relaxation as occurs in children would do just the opposite. (**B**) Schematic showing various measurements from the transmitral flow velocity profile by pulsed wave Doppler at the mitral leaflet tips: LV isovolumic relaxation time (IVRT) between the end of LV ejection or aortic valve closure (AVC) and the start of mitral inflow (typically 70–100 ms), mitral E wave velocity, E wave deceleration time (EDT, normally 160–250 ms), mitral A wave velocity and A wave duration (A dur, normally about 100 ms). The normal E/A velocity ratio in adults is typically between 1 and 1.5. In children, it can be >10, resulting physiological S3, and in elderly < 1, resulting in physiological S4. MV = Mitral valve flow.

The transmitral flow is recorded at the tip of the mitral leaflets using a small sample size (2–3 mm) of pulsed wave Doppler to obtain a pure signal at that point, optimizing the filter and gain to see the profile clearly down to the baseline without noise. This will help obtain an accurate E wave deceleration time and A wave duration. This should not be obtained either distally or proximally, as the profile varies and the profile at the annulus is only obtained for volumetric purposes. A distal sample will provide a different profile, which is slightly delayed without augmentation from flow-dependent mitral leaflet opening. Figure 8A,B show an illustration of the genesis of mitral flow and various measures that can be obtained.

Table 6 lists various factors that can affect the LV isovolumic relaxation time, E wave amplitude and A wave amplitude. It is important to consider these factors and integrate them with other measures of diastolic function.

#### 3.2.2. Pulmonary Vein Flow

**Figure 9:**
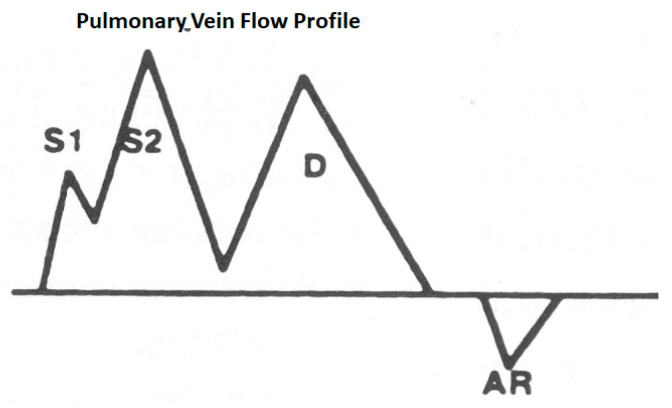
Schematic of pulmonary vein flow: S1 is a systolic wave produced because of left atrial relaxation causing a suction force, S2 is a systolic wave because of mitral annular descent stretching the left atrium and also right ventricular ejection transmitted through a low resistance, short pulmonary circulation D wave because of left atrial emptying when the mitral valve opens and the AR wave, which is a reflux into the pulmonary vein because of left arial contraction. Generally, the S waves are taller than the D waves, the transitions are smooth and the AR wave duration is a measure of the duration of left atrial systole, which is normally about 100 ms.

On transthoracic echo, this is typically obtained from the apical view and from the right upper pulmonary vein. A 2–3 mm pulsed wave Doppler sample should be placed inside the vein, guided by color, to obtain a pure profile without noise from the tissues. During transesophageal echocardiography, it can be obtained from all four veins, and the profiles would vary and would depend on the body position as well. Figure 9 shows such a profile and describes its genesis.

**Figure 10:**
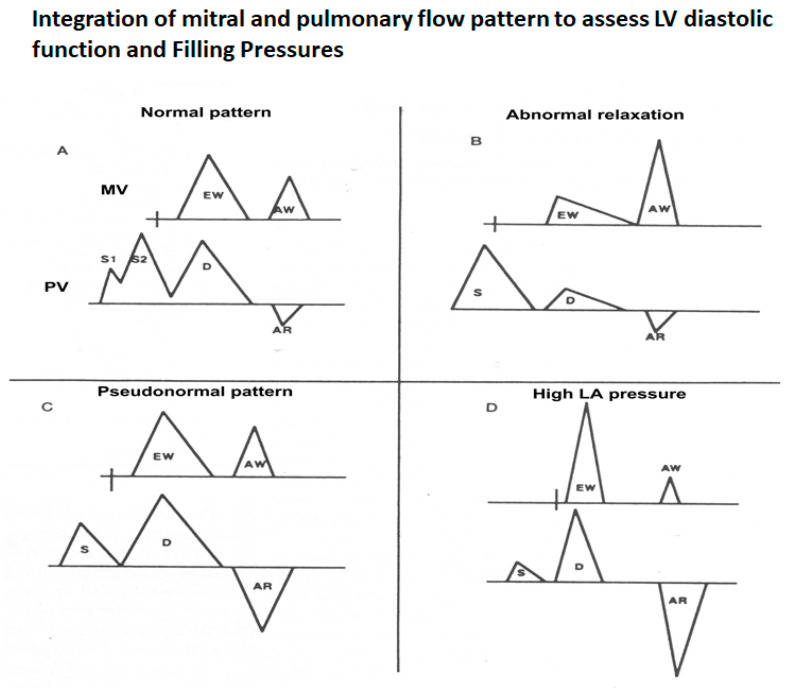
Schematic that shows how to interpret the mitral and pulmonary vein flows as a unit. (**A**) Normal pattern–IVRT 70–100 ms, E/A velocity ratio 1.0 to 1.5, E wave deceleration time 160–250 ms, S wave higher than D wave and AR wave duration same or less than that of the A wave duration. (**B**) Pattern of abnormal LV relaxation–IVRT >100 ms, E/A ratio <1, E wave deceleration time >250 ms and more prominent S wave because of the diminutive D wave secondary to the smaller E wave. (**C**) Pseudonormal pattern that is a combination of impaired LV relaxation and elevated left atrial pressure—these two work in opposite directions to normalize the mitral flow. But the clue is changes in pulmonary vein flow—mainly, the AR wave duration, which will be greater than the mitral A wave duration by >30 ms, indicating longer atrial systole, a marker of elevated LV end-diastolic pressure. Reduced LA compliance may also reduce the S wave amplitude. (**D**) Severely elevated LA pressure characterized by short IVRT (<70 ms), E/A velocity ration >2, E wave deceleration time <150 ms, S wave shorter than D, D wave deceleration time <170 ms and exaggerated AR wave duration and amplitude.

Figure 10 illustrates how the mitral and pulmonary vein flows can be used to characterize the LV diastolic function and filling pressures. This, combined with the mitral annular velocities, provides powerful insights into the LV diastolic performance.

#### 3.2.3. Doppler Tissue Imaging (DTI)

**Figure 11:**
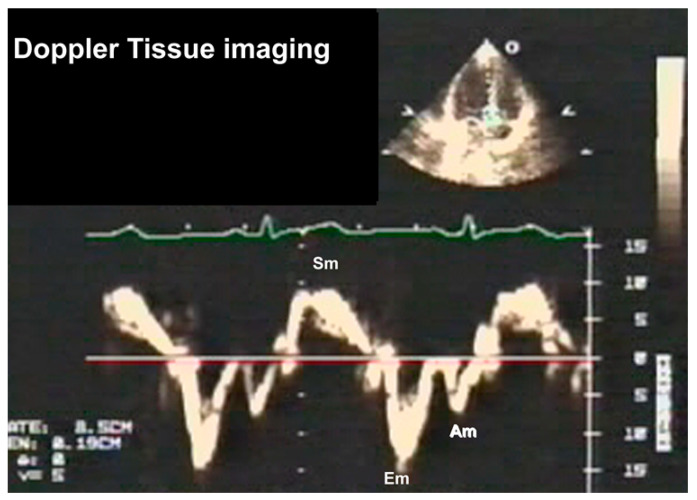
Components of mitral annular velocities obtained by Doppler tissue imaging from the LV apical view. The Sm wave is produced by the mitral annular descent, resulting in this apically directed velocity, the Em or E’ early diastolic velocity, which is produced by mitral annular ascent because of LV relaxation and is a good index of rate of LV relaxation and the Am or A’ velocity because of atrial contraction causing further annular ascent. The E’ velocity varies by the mitral annular quadrant and is generally >8 cm/s for the medial mitral annulus and >10 cm/s for the lateral annulus. Impaired LV relaxation causes a reduction in these velocities. Regional abnormalities will affect these regionally. In constrictive pericarditis, where the lateral LV wall is tethered to the pericardium, the lateral annular E’ velocity will be lower than the medial E’ velocity as the medial annulus compensates for the restricted LV lateral wall long axis function.

**Figure 12:**
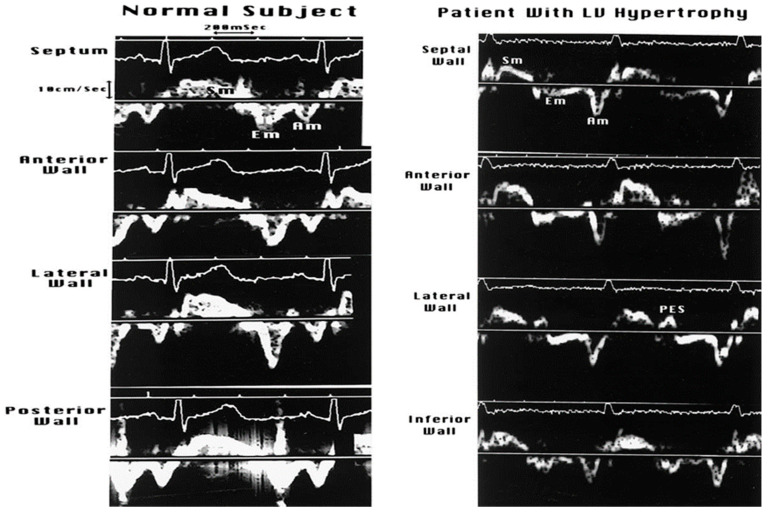
In normal hearts, there is striking synchrony of the contraction and relaxation of various myocardial walls, both in systole and diastole, in terms of the onsets and offsets of these mechanical events. This synchrony is lost in myocardial disorders, including LV hypertrophy. Diastolic asynchrony is one of the bases of abnormal LV relaxation. Systolic asynchrony reduces the coordination of LV power generation as well.

**Figure 13:**
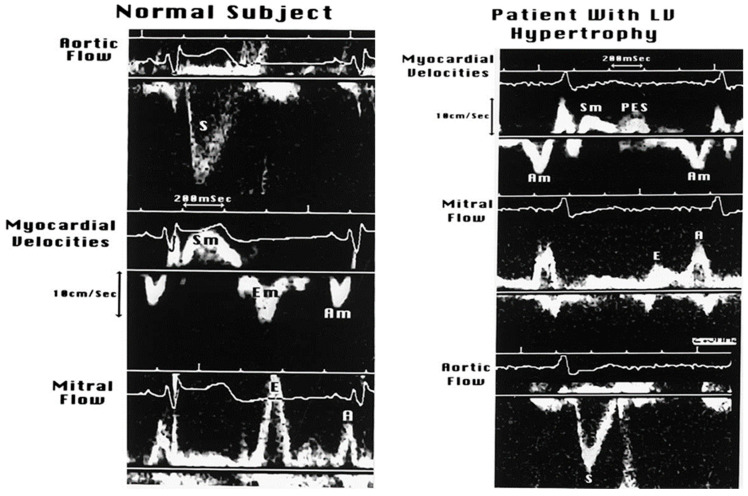
In normal subjects, the LV ejection starts within 10 ms of coordinated shortening of the LV wall segments and the mitral E wave follows the annular E’ wave within 20–40 ms. In those with LV hypertrophy, the LV systolic and diastolic dyssynchrony impairs both LV systolic and diastolic functions. Post-ejection LV shortening (PES) is common in such patients and markedly impairs the pressure decay in LV or LV relaxation.

Traditionally, these velocity profiles are obtained from the medial and lateral mitral annulus using pulsed wave Doppler technology, setting the bandpass filter to capture only low-velocity, high-amplitude signals because the annular velocities are in about the 5–20 cm/s range and the signals are very intense, unlike the blood pool signals produced by moving red blood cells [16]. Figure 11 describes these signals and their genesis. In a normal heart, there is striking synchrony of these signals obtained from different quadrants of the mitral annulus and these are closely linked to both LV filling and ejection functions, as shown in Figure 12 and Figure 13. In myocardial disease, there may be dyssynchrony or incoordination among different myocardial walls, leading to the impairment of both systolic power generation and relaxation because of the incoordination in time and space. The annular e’ velocity is a good marker of LV relaxation and is reduced in impaired relaxation. The mitral E/annular e’ velocity ratio is a good reflection of the mean left atrial pressure, where a ratio ≥15 reflects elevated LA pressure.

**Figure 14:**
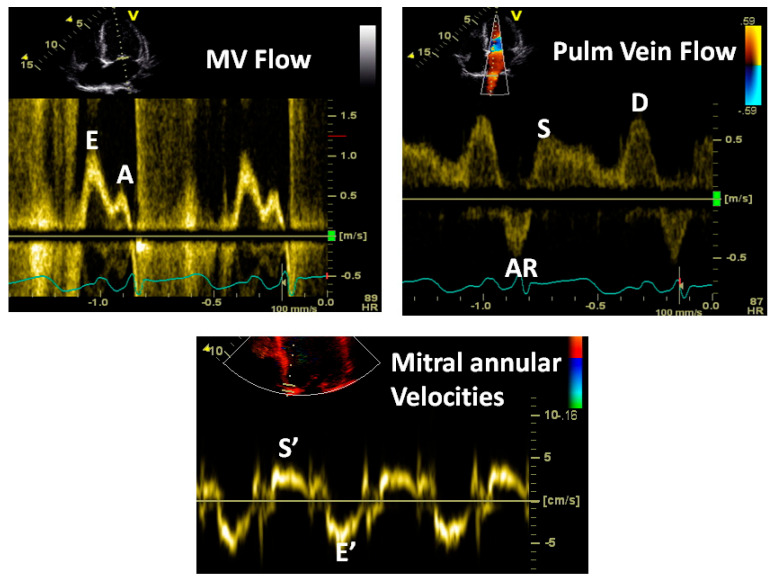
Note the classic pattern of increased LA pressure: E/A velocity ratio >2, short E wave deceleration, E/E’ ratio >15 and an AR wave duration more than the mitral A wave duration. The last measure indicates elevated LV end-diastolic pressure and stiffness.

**Figure 15:**
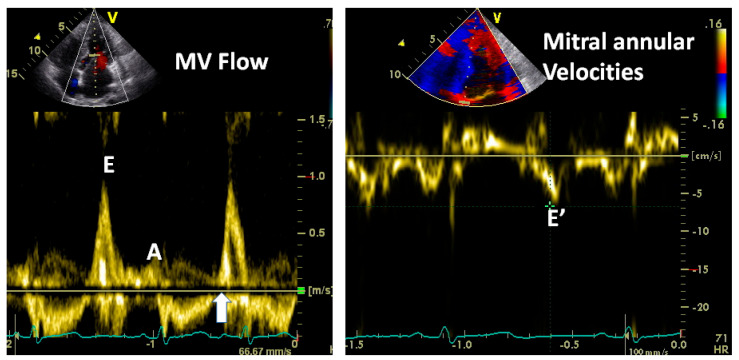
Note that this patient has very high LA pressure signified by a high E/A velocity ratio and high E/E’ ratio. But note that that the IVRT (vertical arrow) is practically zero, indicating that the mitral valve opens immediately after aortic valve closure. In other words, the left atrial V wave is as high as the aortic end-systolic pressure (80–90 mmHg), indicating severe mitral regurgitation. A mean LA pressure in that range is incompatible with life.

**Figure 16:**
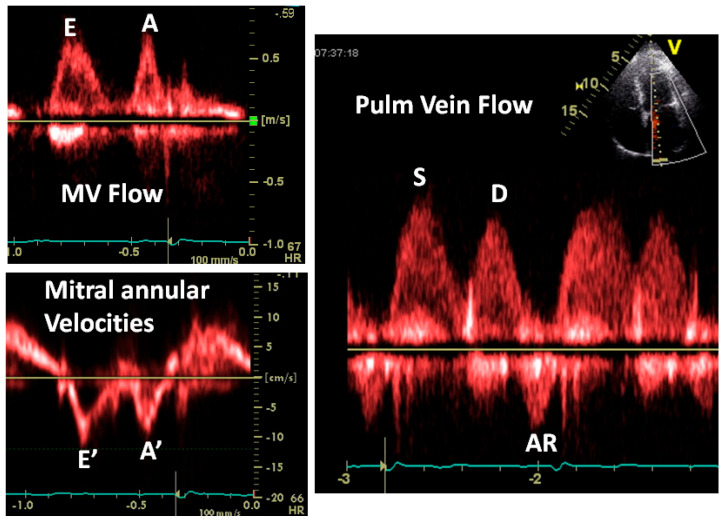
Not everything is “black and white”. In this example, the mitral flow and annular velocities look normal. The E/E’ ratio is normal. The S wave is larger than the D wave and you may conclude that the LA pressure is normal. But note 2 things: the pulmonary AR wave duration is greater than that of the mitral A wave, indicating elevated LVEDP and the sharp transition between the S and D waves, indicating a stiff left atrium, a marker of elevated LA pressure. The shortness of breath in this patient was due to “diastolic heart failure” verified by cardiac catheterization.

Figure 14, Figure 15 and Figure 16 show some examples of how to combine these measurements and also cover some nuances concerning how to interpret these signals.

#### 3.2.4. Left Atrial Volume and Dynamics

The left atrium can dilate because of atrial fibrillation, atrial myopathy or chronically elevated left atrial pressure [17,18]. In other words, an increased LA volume as a marker of chronic LA pressure elevation is comparable to “HbA1C” as a marker of hyperglycemia over a period of time. If the operative left atrial pressure is high, it operates at the steeper portion of the left atrial pressure–volume curve and fills only a little during systole when the mitral valve is closed and the LA receives blood from the pulmonary veins. The higher the LA pressure, the less the systolic filling of LA and the chamber enlarges minimally. Consequently, the passive LA strain will be reduced and the LA “ejection fraction” will be lower. 

#### 3.2.5. Mitral E and A Wave Propagation in LV and Their Changing Profiles as Measures of LV Relaxation and Stiffness 

Slower mitral E wave propagation into the LV correlates with reduced LV relaxation and faster mitral A wave propagation into LV outflow tract accompanied by its reduced duration correlates with late diastolic LV stiffness [19], but these are somewhat cumbersome to use. These have been supplanted by mitral annular velocities, which provide valuable information about the LV diastolic function a lot more easily.

### 3.3. Assessment of Myocardial Structure

The ultimate goal of all these exercises is to find out what is happening in the myocardium in an effort the retard or reverse the process that has been causing LV diastolic function. However, this area is still quite primitive. Cardiac MRI is perhaps the most valuable tool for characterizing the myocardium as it can provide insights into the wall thickness, fibrosis, patten of fibrosis, edema, lipid or iron contents and extracellular volume [20,21,22,23]. Cardiac CT HU units can offer some insights as a normal myocardium is about 40–60 HU, fatty metaplasia will be much lower in the negative range, and fibrosis in 60–100 HU units [24,25]. Intramyocardial calcification can be 100–400 HU. Acoustic elastography has been used to measure the stiffness of the liver, but it has not worked well for the heart [26,27]. Integrated backscatter analysis has the potential to differentiate normal and viable muscle from fibrous tissue and other infiltrates [28], but these ultrasound applications are far from routine in clinical practice. The application of AI may perhaps help advance this field.

## 4. Summary and Conclusions

We have reviewed the clinical relevance and importance of LV diastolic function, the principles behind its assessment and the tools available to assess these in practical terms. Further work in this area and in the field of myocardial tissue characterization will be very important to understand, diagnose, prevent and treat the syndrome of “diastolic HF” or HFpEF. Our goal was to simplify and convey these concepts effectively rather than to perform an in-depth analysis of all the existing literature. We have not delved into the evolving thoughts on treating “diastolic HF”. We would encourage readers to view these publications to gain insights into these [29,30,31,32]. 

## Figures and Tables

**Table 1:** History of concept developments in terms of diastolic HF.

100 AC	Galen proposed that the heart filled by dilation of the right ventricle
1628	Harvey proposed that most ventricular filling occurred during atrial systole
1877	Francois-Franck proposed that most LV filling occurred in early diastole
1906	Henderson described three phases of diastole
1921	Wiggers and Katz reported that the atrial contribution to LV filling varied
1927	Meek proposed that the “contraction remainder” extended into diastole
1949	Wiggers coined the term “inherent elasticity” to describe the diastolic properties of the myocardium
1970s	Studies were performed on LV relaxation and passive LV properties, and these were characterized
1970s	Extensive studies of hypertrophic cardiomyopathy describing severely abnormal diastole and potential benefits of verapamil
1980s	Diastolic heart failure was described as an entity
1992	Monograph on “Diastolic heart failure” published by Shah and Pai
2000s	The term “Heart failure with preserved ejection fraction (HFpEF)” was coined because of uncertainties about evaluating LV diastolic function

**Table 2:** Factors affecting LV relaxation.

**Factors impairing LV relaxation:**
Aging
LV hypertrophy
Myocardial ischemia
LV dyssynchrony; e.g., RV pacing, LV hypertrophy, LBBB
Myocardial disarray as in hypertrophic cardiomyopathy
Increased LV afterload causing increased myocyte calcium load
**Factors that improve LV relaxation:**
Higher sympathetic tone and catecholamines
Calcium channel blockers
Specific LV lusiotropic agents such as levosimendan and calcium channel blockers in hypertrophic cardiomyopathy

**Table 3:** Processes associated with increased LV diastolic stiffness.

Increasing age
LV hypertrophy
Interstitial fibrosis
Myocardial scarring
Infiltrative disorders such as cardiac amyloid, sarcoid and other storage disorders

**Table 4:** Diastolic heart failure mimickers.

Pericardial constriction
Acute pericarditis
Acute aortic regurgitation
Acute mitral regurgitation
Acute tricuspid regurgitation
Right ventricular infarction
Acute massive or submassive pulmonary embolism
Large pleural effusions
Tension pneumothorax
Large mediastinal tumors
Use of positive end-expiratory pressure (PEEP) and auto PEEP

**Table 5:** Commonly used terminology addressing LV diastolic function with the relevant definitions.

Force:	An agent that causes a change in momentum
Stress:	Force per unit cross-sectional area that causes deformation
Strain:	Fractional change from unstressed dimension
Creep:	Time-dependent elongation of a material held at constant stress
Stress relaxation:	Diminution of stress when held at constant strain
Elasticity:	Property of recovery of a material from a stressed state when the stress is removed
Viscosity:	Property of the material that retards deformation in response to stress
Viscoelasticity:	Property of the material when stress depends upon strain, e.g., myocardium
Hookean material:	Material that follows Hooke’s law, which states that the stress–strain relationship is linear
Non-Hookean material:	Material that does not follow Hooke’s law, e.g., myocardium
Modulus of stiffness:	Slope of the stress–strain relationship
Compliance:	Change in volume per unit change in pressure for a chamber
Stiffness:	Reciprocal of compliance

**Table 6:** Factors affecting LV IVRT, E wave amplitude and A wave amplitude.

**Factors affecting LV IVRT:**
Rate of LV relaxation increases with impaired relaxation
LBBB increases IVRT
RV pacing increases IVRT
High LA pressure reduces IVRT
Large left atrial V wave reduces IVRT
**Factors affecting E wave amplitude:**
Impaired LV relaxation reduces E wave amplitude
High LA pressure increases its amplitude
Exaggerated LV recoil increases its amplitude
**Factors affecting mitral A wave amplitude:**
Left atrial contractile function: reduced in atrial cardiomyopathy, post cardioversion
Atrial preload: larger LA volume at the time of systole boosts the atrial transport function
Atrial afterload: Higher LVEDP or pre-A pressure will reduce atrial contribution
